# Using LDR as Sensing Element for an External Fuzzy Controller Applied in Photovoltaic Pumping Systems with Variable-Speed Drives

**DOI:** 10.3390/s150924445

**Published:** 2015-09-22

**Authors:** Geraldo Neves De A. Maranhão, Alaan Ubaiara Brito, Anderson Marques Leal, Jéssica Kelly Silva Fonseca, Wilson Negrão Macêdo

**Affiliations:** 1 Departamento de Ciências Exatas e Tecnológicas, Universidade Federal do Amapá (Unifap), Rod. Juscelino Kubischeck, km 02, Jardim Marco Zero, Macapá, Amapá 68903-419, Brazil; E-Mail: aubrito@unifap.br; 2 Instituto de Tecnologia, Universidade Federal do Pará (UFPA), Rua Augusto Corrêa, 01, Guamá, Belém, Pará 66075-110, Brazil; E-Mails: sndersonleal@gmail.com (A.M.L.); jessicakellyfonseca@gmail.com (J.K.S.F.); wnmacedo@ufpa.br (W.N.M.)

**Keywords:** fuzzy controller, photovoltaic system, photovoltaic water pumping, variable-speed drive

## Abstract

In the present paper, a fuzzy controller applied to a Variable-Speed Drive (VSD) for use in Photovoltaic Pumping Systems (PVPS) is proposed. The fuzzy logic system (FLS) used is embedded in a microcontroller and corresponds to a proportional-derivative controller. A Light-Dependent Resistor (LDR) is used to measure, approximately, the irradiance incident on the PV array. Experimental tests are executed using an Arduino board. The experimental results show that the fuzzy controller is capable of operating the system continuously throughout the day and controlling the direct current (DC) voltage level in the VSD with a good performance.

## 1. Introduction

A Variable-Speed Drive (VSD) is an electronic device that allows power supply with variable voltage and frequency. This equipment was developed for the speed control of electric motors using alternating current (AC), particularly for the three-phase induction motor.

Controlling the induction motor speed means controlling the power consumed by it. This functionality has made VSD an excellent technical and economic option for direct-coupled photovoltaic water pumping system applications, without using energy accumulators.

In this type of application, the VSD used must necessarily have the Proportional-Integral-Derivative (PID) controller embedded in its physical structure. This controller acts as the “brain” of the system, allowing the VSD to deliver power to the load (pump) according to the power available from the photovoltaic (PV) array, *i.e.*, the higher the power available from the source, the greater the volume of water pumped by the system.

This application is well established in the scientific community with several published studies [1,2,3,4,5,6,7]. The most important point regarding VSD programming is the PID controller’s exact tuning, commonly accomplished by a trial-and-error method, because the system transfer function is unknown [4]. Most modern drives have the possibility to identify these parameters automatically and to tune the parameters of controller. In this type of application less modern VSDs are used in order not to increase the cost of the photovoltaic pumping system. These VSDs do not have the PID auto-tuning feature. In addition to this, despite the VSD and pumps having known models, the system transfer function (generator PV + VSD + pump) is dynamic and varies according to the power delivered by the photovoltaic generator and the height of the column of water in which the pump operates. A systematic procedure consists in experimentally obtaining the system transfer function where the PID tuning parameters (proportional gain, integral time, and derivative time) are subsequently determined [4].

However, not every VSD has an embedded PID controller, for example those available in the 127 V (voltage AC) voltage range. The use of a VSD in the 127 V voltage range has the advantage of enabling less use of photovoltaic modules in series; half of what is commonly used when there are frequency inverters in the 220 V voltage range. This ensures that, even in low-power systems, it is possible to use high-power modules, implying the use of modules with better performance, as well as a lower cost for watt-peak (Wp).

Considering the difficulty in tuning the PID controller, as well as providing the use of VSDs that do not have it embedded, this paper proposes the development of a control system based on Fuzzy Logic Systems (FLS), able to maintain a stable DC excitation voltage level of the VSD.

Controlling the direct current (DC) bus voltage level is necessary to ensure the availability of Photovoltaic Pumping Systems (PVPS), avoiding interruptions in system operation caused by undervoltage errors in the VSD. The VSD is parameterized to provide the pump operating rated frequency, but an unwanted reduction in the DC bus voltage level occurs when the power supplied by the PV generator does not correspond to enough power to operate the pump at rated frequencies [4].

The fuzzy controller is able to maintain the voltage level of the DC bus close to the reference value by adjusting the pump operating frequency, considering the irradiance variations that the PV generator submits.

It should be emphasized that the fuzzy logic system (FLS) enables advances in the development of maximum power point tracking (MPPT), which is justified by the possibility of FLS operation with a variable setpoint if a sensor with greater precision is used.

The fuzzy controller design is based on a non-linear control method, and the control performance is less affected by the system parametric variation.

Moreover, fuzzy techniques are constituted by a base of linguistic rules, which are composed by exploiting qualitative aspects and expert knowledge regarding the problem. These features eliminate the need for a control plant mathematical model, providing a design procedure and sizing of the simplest controller to be implemented, even when empirical methodologies for fine-tuning of parameters are employed. Alternative methods based on intelligent systems, such as artificial neural networks, genetic algorithms, and hybrid systems, which are also characterized by the ability to deal with such problems, also have provided promising results [8,9,10,11,12].

This paper is structured as follows: first, the use of the type of Light-dependent Resistor sensor is presented; then, the support platform for programming is approached; after that, the fuzzy controller is presented; next, the developed fuzzy controller is shown; later, experimental results are exposed; and finally, conclusions from the work are drawn.

## 2. Experimental Section

### 2.1. Light-Dependent Resistor

**Figure 1:**
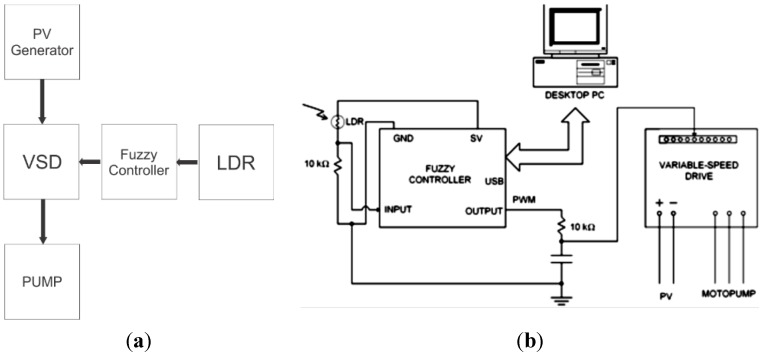
(**a**) Photovoltaic Pumping Systems (PVPS) Block Diagram with External Control; (**b**) Circuit using Light-dependent Resistor (LDR) connected to the fuzzy controller.

A Light-dependent Resistor (LDR) sensor is used to obtain the proportional irradiance value in terms of optical radiation incidence to which the PV generator is submitted. The LDR is an inexpensive cadmium sulfide (CdS) photoconductive cell, in which resistance decreases with the increase of illumination incident on the cell. This type of sensor is commonly used in a resistive arrangement in series, where it can get a voltage value that increases with increasing illumination. Figure 1a shows the block diagram of PVPS, and Figure 1b shows the resistive circuits in series connected to the representative block of a fuzzy controller.

The LDR consists of a low-cost alternative, which has good applicability to the pumping system. Systems, such as calibrated cells or commercial pyrometers, have greater accuracy in measurements, but also a higher cost, which is not desirable. On the other hand, even with less accuracy, the LDR can satisfactorily detect variations in irradiance for qualitative analysis purposes.

The datasheet of the LDR NSL19 RS-M51 features the resistance value variation with the illumination. The range provided by the manufacturer is from 0 to 10,000 lux, with resistance values linearly varying from 1000 kΩ to 100 Ω, respectively. It is necessary to observe the LDR behavior when it is exposed to actual levels of lighting on a sunny day, because in these conditions, the resistance variation is not provided by the manufacturer (values exceeding 10,000 lux).

In order to check the LDR behavior when it is subjected to actual meteorological conditions, a measurement test is executed. Considering an LDR exposed to sunlight for a whole day, the test corresponds to samples performed in 1-s intervals of the input voltage value presented in the circuit from Figure 1.

**Figure 2:**
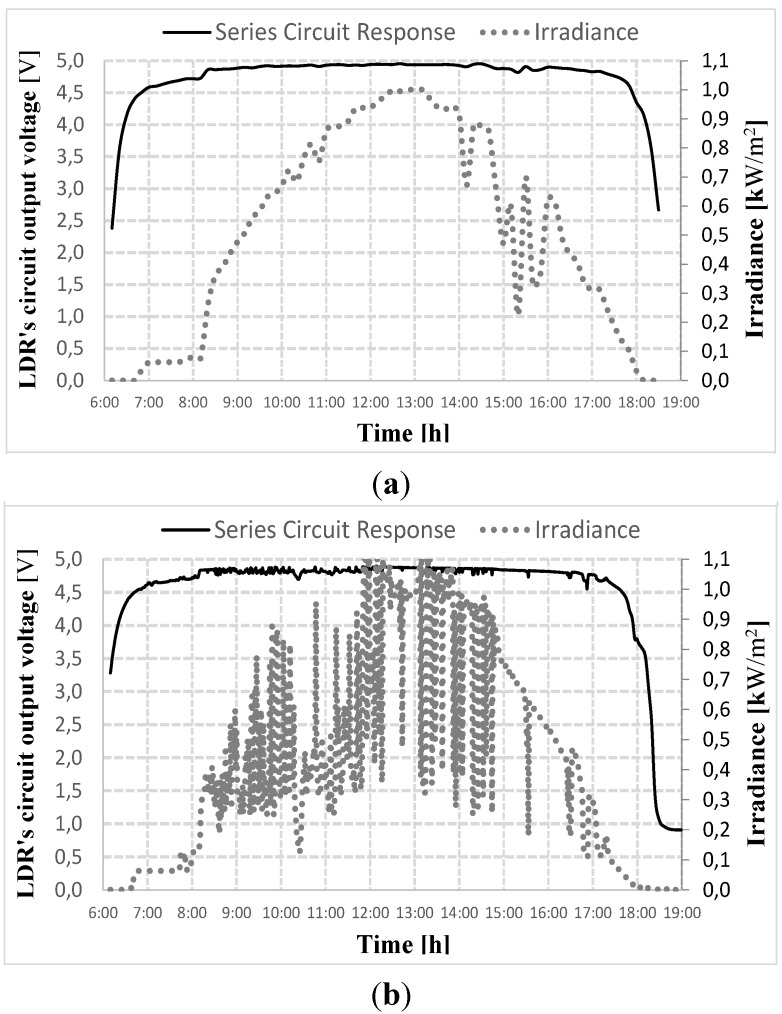
LDR series circuit response and irradiance during the day time, (**a**) sunny day and (**b**) cloudy day.

**Figure 3:**
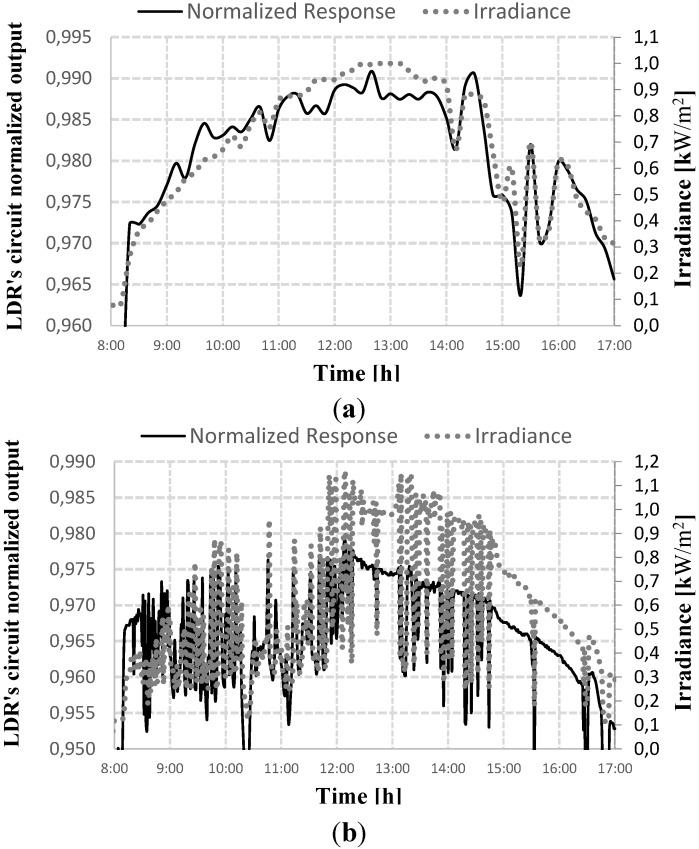
LDR normalized response, (**a**) sunny day and (**b**) cloudy day.

Figure 2 shows the illumination behavior measured on the LDR circuit on two different days. It can be seen that the LDR, when subjected to high illuminance, operates out of the linearity region provided by the manufacturer, that is, it is not possible to accurately acquire the relationship between the LDR resistance and illuminance, However, it is possible to observe that, even outside the LDR’s linearity region, the LDR circuit response shows a significant sensitivity to illuminance variations. This can most clearly be observed when using the normalized values, as can be seen in Figure 3.

Figure 3a,b shows the normalized values of the LDR’s response for the period between 8:00 a.m. and 5:00 p.m. on two different days. It is emphasized that the normalized values correspond to the voltage supplied by the series circuit shown in Figure 1b. In this case, the base value for the normalization is the maximum possible voltage value in this arrangement, namely 5 V. It can be seen that the LDR is able to provide information about the increase or decrease in illumination, which is equal to the increase and decrease of the power supplied by the PV generator throughout the day. It should also be noted, in Figure 3, that the LDR series circuit responds adequately to the variations in irradiance, thus, it is able to be use in controlling applied photovoltaic systems.

**Figure 4:**
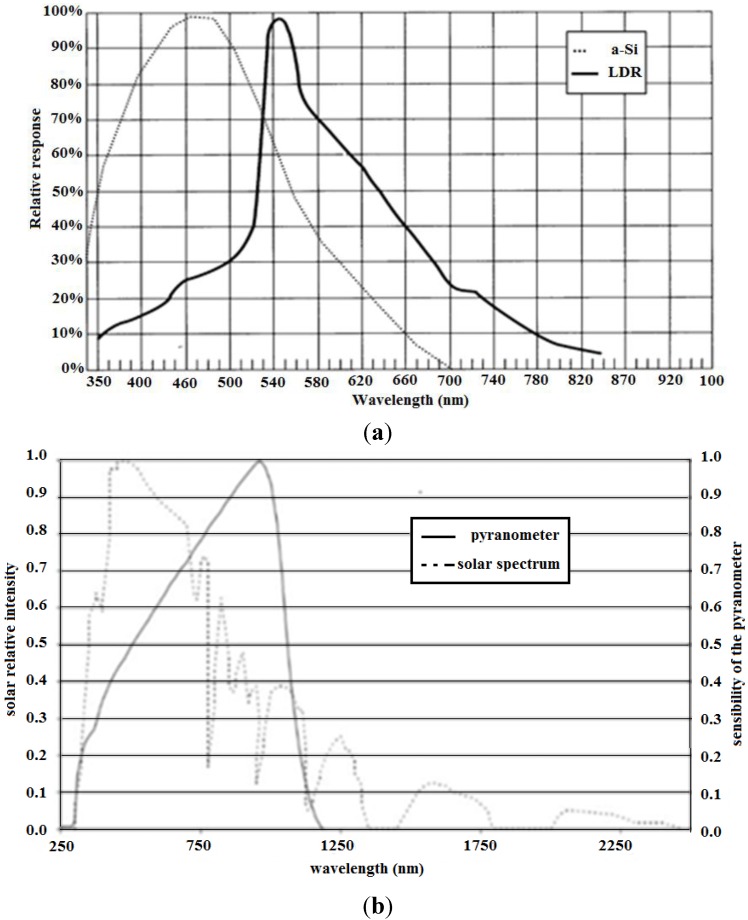
(**a**) The spectral response of amorphous silicon and the LDR (**b**) solar radiation spectrum.

LDR does not have as objective of measuring the irradiance accurately, but rather, only to identify the increase or decrease trend for fuzzy control. Since its spectral response matches significantly with the spectral solar irradiance, the LDR could be used for evaluation of the irradiance variation trend, although it is significantly different from the spectral response of amorphous silicon. The comparison between the solar irradiation spectrum and the spectral response of the LDR and amorphous silicon can be seen in Figure 4.

### 2.2. Fuzzy Controller Device

An Arduino board is an open-source physical computing platform based on a single-board microcontroller, and it was used for programming the fuzzy controller developed here. The hardware consists of an open-source hardware board, designed around an 8-bit Atmel AVR microcontroller, or a 32-bit Atmel ARM.

The ATmega328 program memory (flash memory) is large enough (32 kB) to support the programming of a fuzzy system for application in PVPS. The amount of I/O ports is satisfied since the fuzzy controller does not use a quantity greater than three (3) of these terminals for its operation. The clock frequency of 16 MHz is also supported and suitable for application in PVPS since the changes of the meteorological variables that are detected by the fuzzy controller are from in the order of units of seconds.

The Arduino UNO Rev3 model is used as the fuzzy controller based platform. The Arduino UNO is a low-cost alternative for controllers programming, which uses the Atmega328 processor and board with input and output terminals.

The VSD used here, like other VSDs available on the market, has an auxiliary input which helps to control the pump operating frequency, called Analog Input (AI). The AI regulates the frequency value using an input voltage ranging from 0 to 10 V, which corresponds to the minimum and maximum values for the pump frequency, respectively (0 V corresponds to 0 Hz and 10 V corresponds to 60 Hz). The Arduino output signal, however, uses Pulse-width Modulation (PWM). To use such a signal in the VSD AI, a resistor-capacitor (RC) circuit is required to conditioning of that sign.

The schematic diagram of the VSD control system is presented in Figure 1b. It can be seen the RC circuit, which, according to the values used in the design of the circuit (resistance of 10 kΩ and 10 μF capacitance), its time constant (0.1 s) is not capable of impacting the control signal dominant dynamic of the VSD AI input. A microcomputer is used to acquire the data necessary to verify the fuzzy controller performance.

### 2.3. FLC Description

The proposal of an FLS to control the VSD’s DC bus voltage level in PVPS uses the same methodology employed in FLC drivers for maximum power point trackers [13,14]. The main objective of this controller is adjusting the pump frequency at the worst moments of PV generation in order to ensure the availability of PVPS, reducing undervoltage errors in VSD. The only FLC input is the normalized value of illumination that the LDR submitted (G). The value of the derivative of this normalized value (dG) is calculated in order to detect if there was variation in the power generated by the PV generator.

**Figure 5:**
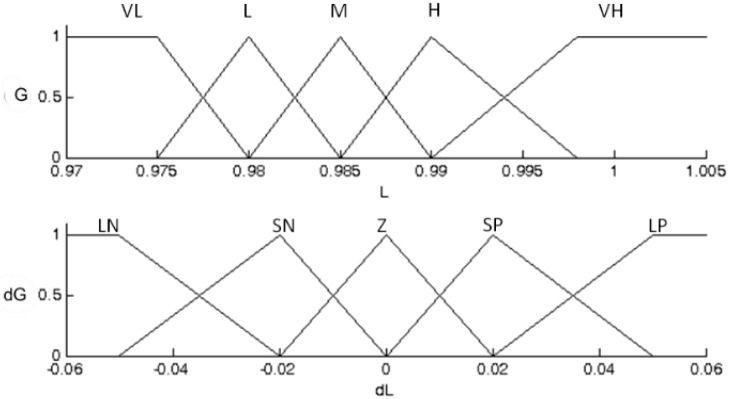
Fuzzy sets input.

Figure 5 shows the fuzzy sets input. The universe of discourse (U) of fuzzy sets corresponds to the region where irradiance is capable of operating the pump without having the undervoltage error. Linguistic variables for G (VH—Very High, H—High, M—Moderate, L—Low and VL—Very Low) and dG (LP—Large Positive, SP—Small Positive, Z—Zero, SN—Small Negative and LN—Large Negative) are defined for use in the system rules base. As can be seen in Figure 5, the membership functions are triangular and trapezoidal.

Based on experimental results, as well as the provisions in [4], it is clear that the pumping period of a PVPS is between 8:00 a.m. and 5:00 p.m. (in days with clear skies). Thus, the FLC actuation period and the fuzzy sets are defined for this day time interval. The value of 0.98, for example, corresponds to the normalized value of the LDR to about 8:30 a.m., which represents a little amount of water being pumped.

**Table 1:** Rule base of fuzzy controller.

	VH	H	M	L	VL
LP	5.0 V	5.0 V	2.0 V	0.5 V	0 V
SP	5.0 V	4.5 V	1.5 V	0 V	0 V
Z	5.0 V	4.0 V	1.0 V	0 V	0 V
SN	2.5 V	1.5 V	0.5 V	0 V	0 V
LN	0 V	0 V	0 V	0 V	0 V

The FLS rule base is shown in Table 1. It can be observed that all the linguistic variables that describe the consequent fuzzy rules correspond to the values of voltage at the output of the controller. These values are responsible for tuning the pump frequency; that is, the displayed voltage value in the rule base equal to 5 V represents the maximum frequency of rotation of the pump (which is 60 Hz). The gain of the AI needs to be set equal to 2 to receive the value of 5 V. The values equal to 0 V are necessary to ensure the stopping of the pump. The relationship between the input linguistic variables of G and dG in the fuzzy rule base was obtained by modeling the knowledge of an expert in PVPS.

Membership functions used in fuzzy sets output are singletons, with center values corresponding to those arranged as linguistic variables, and they are part of the universe of discourse concerning the fuzzy controller output voltage.

The Mamdani implication is used with product t-norm and the fuzzyfication corresponds to the Center Average.

## 3. Results and Discussion

Experimental tests were performed on a workbench for testing pumps, such as the one proposed in [15]. These tests were conducted to evaluate the performance of the fuzzy controller regarding voltage control of the VSD DC bus. During these tests, the monitoring of the DC bus voltage and the normalized response of the LDR throughout the day were carried out. The sample time is 1 min and data storage is performed on a microcomputer. Table 2 describes the test system used.

**Table 2:** Description of test system.

Equipment	Description
Test facility	10 m water column
Motopump	Submersible centrifugal pump (½ HP and 8 stage)
PV generator	12 modules of 45 Wp (Voc = 101 V, Vmp = 75 V, Isc = 0.78 A, Imp = 0.60 A, amorphous silicon). For 3 string parallel strings, each of 4 panels in series
VSD	CFW 08 de ½ HP

**Figure 6:**
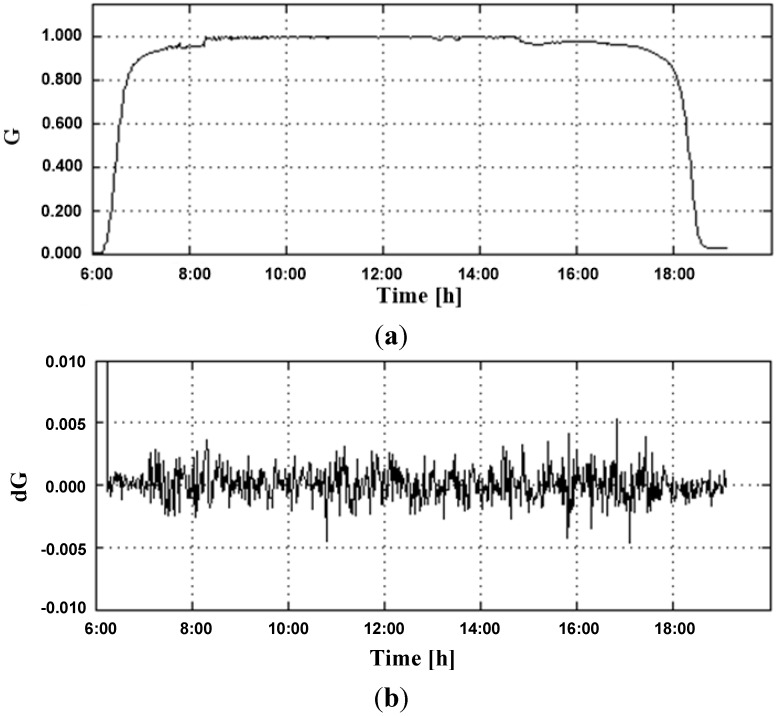
Input signal fuzzy controller (**a**) LDR series circuit response and (**b**) Variation of the LDR series circuit response.

The data acquisition was performed by the same base platform (Arduino) used by the controller. Data storage is done using personal computer, using a USB communication cable and Matlab software. This was necessary because the Arduino’s platform data memory is not large enough for the storage of the samples. Figure 6 presents the input signals of the FLC, where the irradiance variation occurrence, which can cause undervoltage errors in the VSD DC bus, can be observed. It is also possible to note that meteorological variations occurred slowly once an abrupt behavior was not observed in dG.

**Figure 7:**
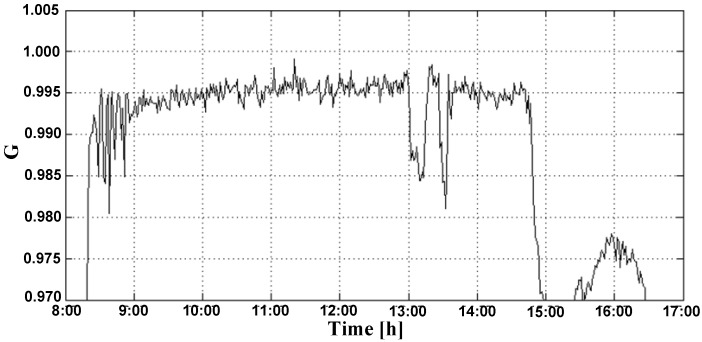
Detailed behavior of the parameter G.

Figure 7 shows the universe of discourse corresponding to the input G in region fuzzy controller. It is possible to see abrupt variations in irradiance on the PV generation time interval.

**Figure 8:**
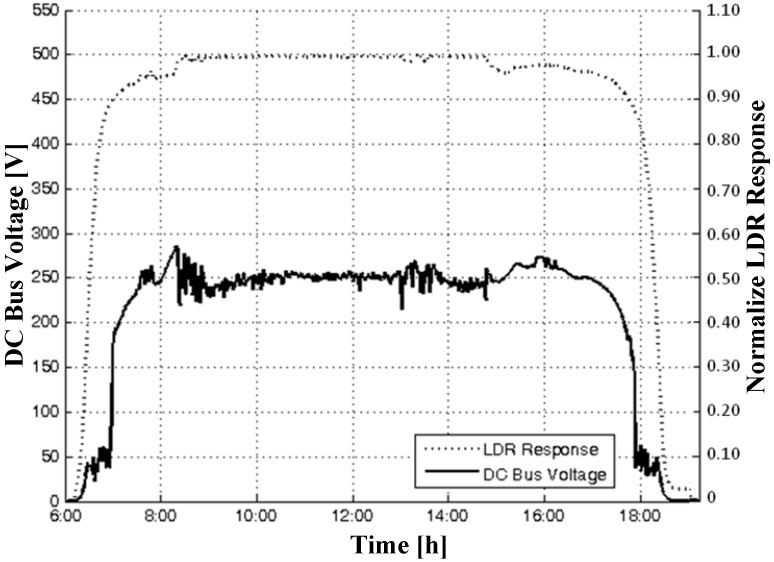
Fuzzy controller performance regarding the DC bus voltage behavior.

Figure 8 demonstrates the fuzzy controller performance considering the DC bus voltage behavior with changes in the irradiance during the day. It can be seen that, at times when there is a steeper variation in irradiance, for example, 8:00 a.m. and 5:00 p.m., the fuzzy controller operates the decelerating of the pump. This is to ensure that the voltage level of the DC bus does not undergo a sudden sinking, resulting in undesired undervoltage errors in the VSD. Thus, the fuzzy controller can ensure the availability of the PVPS, maintaining the operating voltage of the DC bus in a safe operating region, with an average value of close to 250 V during the daytime hours, in which the PV provides enough power to operate the pump.

The value of 250 V was used because amorphous silicon PV modules degrade over time. In this particular case, a reduction of approximately 12.5 V for each PV module on maximum power voltage was verified. Thus, the value of 250 V corresponds to the voltage that most closely matches the maximum power voltage value of the PV generator throughout the day. Thus, for the photovoltaic generator used in this work, the voltage is obtained by the combination of four PV modules in a series circuit (4 × 62.5 = 250 V).

It is also observed that small variations in LDR voltage can represent significant variations in the DC bus voltage. Based on the fuzzy controller operating region, as it is shown in Figure 7, LDR voltage sags, as occurred at 3:00 p.m., causing a significant control signal reduction and making the DC bus voltage float in its open circuit value.

**Figure 9:**
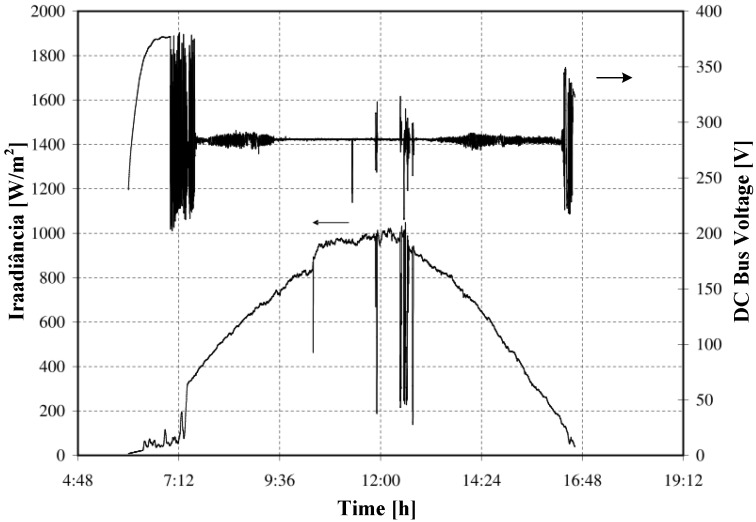
Variation in DC voltage in a PVPS with VSD containing an embedded PID.

A serious problem facing the use of VSD for PV applications is the occurrence of switchings in their power circuit in the daytime hours of lower irradiance. Such a problem does not happen with the FLC system, as is observed in Figure 8. Experimental results, published by [15], show that the PID controller used, contained in many VSDs on the market, to perform the control of the DC bus voltage level, presents inappropriate behavior because the VSD uses fixed voltage on the DC bus, *i.e.*, successive switchings in the daytime hours of lower irradiance, as shown in Figure 9.

These successive switchings can cause permanent damage to the internal circuitry of the VSD. They are caused by the new dynamics of the system imposed by the PID controller. This fact makes the proposed FLC most appropriate for PVPS, taking into account that it eliminates such types of switchings, preserving the VSD circuits.

It is important to emphasize that the use of the Arduino board significantly reduces the cost of implementation of this monitoring and control system. The use of VSD in the external controller using the Arduino board also enables the monitoring of this type of experiment without the need to use specific equipment for data acquisition. This brings a significant reduction in costs related to this experiment.

## 4. Conclusions

In this paper, a fuzzy-based control system to control the DC bus voltage of a VSD applied to PVPS was proposed. Experimental tests were executed to observe the fuzzy controller performance. According to the obtained results, the fuzzy controller performance and the control voltage level of the VSD DC bus were satisfactory. The fuzzy controller has ensured the availability of pump operation by suppressing undervoltage errors in VSD and maintaining the voltage level in an appropriate region of operation, even with the abrupt changes in the level of solar radiations to which the system was subjected.

The elimination of successive switchings in VSD during daytime hours with low levels of irradiance is an important ability of the fuzzy controller when compared to the PID controller characteristics. The absence of such switching ensures a safer operation of VSD as the abrupt changes in voltage, resulting from the dynamics of the PID controller, may cause irreversible damage to the VSD circuits.

It is emphasized that there is also the possibility of implementing the control system proposed in this paper in VSDs that do not have embedded PID controllers. There are some VSDs commercially available on the market that does not have the PID controller incorporated in its physical structure. One example is the equipment sold in the range of 127 V AC. The use of this type of converter has the advantage of enabling the use of less PV modules in series, thereby reducing the cost of implementing the system for small applications (less than 500 Wp installed). In these cases, it also avoids the use of photovoltaic modules with lower power (<50 Wp).

Finally, it is important to mention that, despite the promising results obtained in this work, there is still a need to evaluate the FLS in actual operating real conditions in order to prove its reliability.
